# High-Resolution Representation for Mobile Mapping Data in Curved Regular Grid Model

**DOI:** 10.3390/s19245373

**Published:** 2019-12-05

**Authors:** Jingxin Su, Ryuji Miyazaki, Toru Tamaki, Kazufumi Kaneda

**Affiliations:** 1 Graduate School of Engineering, Hiroshima University, 1-4-1 Kagamiyama, Higashi-Hiroshima, Hiroshima 739-8527, Japan; tamaki@hiroshima-u.ac.jp (T.T.); kin@hiroshima-u.ac.jp (K.K.); 2 Faculty of Psychological Science, Hiroshima International University, Gakuendai, Kurose, Higashi-hiroshima, Hiroshima 555-36, Japan; r-miyaza@hirokoku-u.ac.jp

**Keywords:** road surface, point cloud, mobile mapping system, bilinear interpolation, curved regular grid, OpenCRG

## Abstract

As mobile mapping systems become a mature technology, there are many applications for the process of the measured data. One interesting application is the use of driving simulators that can be used to analyze the data of tire vibration or vehicle simulations. In previous research, we presented our proposed method that can create a precise three-dimensional point cloud model of road surface regions and trajectory points. Our data sets were obtained by a vehicle-mounted mobile mapping system (MMS). The collected data were converted into point cloud data and color images. In this paper, we utilize the previous results as input data and present a solution that can generate an elevation grid for building an OpenCRG model. The OpenCRG project was originally developed to describe road surface elevation data, and also defined an open file format. As it can be difficult to generate a regular grid from point cloud directly, the road surface is first divided into straight lines, circular arcs, and and clothoids. Secondly, a non-regular grid which contains the elevation of road surface points is created for each road surface segment. Then, a regular grid is generated by accurately interpolating the elevation values from the non-regular grid. Finally, the curved regular grid (CRG) model files are created based on the above procedures, and can be visualized by OpenCRG tools. The experimental results on real-world data show that the proposed approach provided a very-high-resolution road surface elevation model.

## 1. Introduction

The modeling of road scenes has become an increasingly important topic in academia and industry. While various techniques have been developed to handle different types of issues, it is recognized that efficiency and reliability are both key considerations in the assessment of systems. However, to help with vehicle development and evaluation, a high-accuracy and high-precision three-dimensional model of the road surface is necessary and very valuable. There are multiple methods of building detailed representations for the road model, and they can result in different models based on various purposes. For example, in [1,2], the authors presented approaches for grid-based road model estimation for advanced driver assistance systems. Their measurements from sensors are transformed into a grid-based road model and a geometrical description is extracted out of this model by the use of a path-planning based method. In [3], a modeling of the road with geometric parameter representation was proposed, which contained three parts of road: straight line, circular arc, and clothoid. The relationship between trajectory curvature and velocity was established, and then a simulation was carried out to verify the road model. It proved that the model could be used to control a four-wheeled robot. For other uses, some authors proposed methods using the elevation information to building road models [4,5,6,7]. It has been proven that the elevation-based methods are suitable for detection techniques.

However, precise 3D road surface measurements and efficient 3D road data representation are two essential requirements for building high-precision 3D road models. To fulfill the requirement of precise road surface measurements, we use a point cloud as input data. Point cloud data is comprised of a set of measured points in three-dimensional space. The point cloud is one of the most widely used data types in three-dimensional image processing. Point clouds are already having a huge impact on different industries, especially on road-related projects (e.g., road surveys, road modeling, and autonomous vehicles). Mobile mapping systems are one of the most widely used surveying devices for capturing large-scale point clouds and digital images. Authors in [8] provided a recently available review of mobile mapping system (MMS) and surveying technologies. Some of the newly developed and presented systems are described in [9,10,11,12]. These systems produce large-scale 3D point clouds and very-high-precision geometric measurements. The produced point clouds are used for many road-related research tasks, including missing road point regions detection [13], road damage information detection [14,15], road segmentation and recognition [16,17], etc.

Since the geometric relationships between road segments are normally assumed as straight line segments and curved arc segments, there are many different approaches that are available, dealing with various situations. Examples can be found in [18,19,20,21]. In geometric road design, G2 continuity is demanded, which means that not only the tangent vectors between different road segments are lying along the same direction, but also have the same curvature at the joint point. The commonly used algorithms for straight line and circular arc detection are the random sample consensus (RANSAC) and the Hough transform. A comprehensive overview of recent research in RANSAC-based estimation methods is given in [22]. Therefore, most strategies focus on the transition curves. In [23,24], the authors demonstrate a workflow for representing point cloud data in a curved regular grid model. The input laser-scanned point clouds and geometric description of the road both need strong manual intervention in the preparation phase. The selected pilot road has a near perpendicular segment, and the elevation values are calculated by a fixed-radius nearest-neighbour search algorithm from the input point cloud directly. The mean elevation values of points inside the circle are stored in the curved regular grid (CRG) cells. This also causes the problem that the resolution of the generated CRG model is limited by the density of the input point cloud. However, the performance and accuracy of the two key steps—road segmentation and elevation estimation—can be further enhanced. In our previous work [25,26,27], we presented a workflow which can produce a high-precision three-dimensional point cloud model of a road surface region and trajectory points. In this paper, we extend the method based on our previous results for representing the mobile mapping data in the CRG model efficiently.

In this work, the goal was to build a road model that contains geographic information of the road surface and use elevation information to show the shape of the road surface. We first apply a robust and effective method that can divide the road into three road segment categories: straight line, circular arc, and clothoid curve. For the straight line and circular arc solutions, we utilize the RANSAC algorithm. Then, we adopt a G2 interpolation method [28] to estimate the transitions between road segments, that is, the clothoid curves. To create a CRG model file, a regular grid which provides elevation values is needed. To effectively and accurately accomplish this, we use a two-step method to generate the regular grid. In the first step, for finding the regular grid, a non-regular grid is initially created from the input point cloud and trajectory points. In the next step, we estimate the regular grid from the non-regular grid by applying a bilinear interpolation method. Finally, the presented process is applied to our real-world point cloud data collected from the Japanese highway network.

In sum, the main contributions of this work are as follows. First, in the data preparation phase, the whole process operates without any human intervention. To enhance the accuracy of elevation estimation, with the two-step method, the bilinear interpolation ensures the elevation values are precisely computed. It can also provide a guarantee of high resolution, where the grid resolution can be selected by the user. Secondly, to improve the time and space efficiency compared with the nearest-neighbour search approach used in [23], the two-step method can decrease the storage and query execution time. Furthermore, our previous work results provide precise road surface region point cloud and trajectory points. Precision data can be used to create a more accurate elevation regular grid for the purpose of building the CRG model. Finally, with the help of OpenCRG, we can represent three-dimensional (3D) road data in CRG models.

### 1.1. Overview of OpenCRG

In 2005, a project called OpenDRIVE [29] was started by a team of driving simulation experts from Daimler AG and VIRES Simulationstechnologie GmbH. This project aimed to standardize road description in order to facilitate the data exchange between various driving simulators. This is the first member of the OpenSolutions family. After the debut of OpenDRIVE in 2006, other big companies joined in (e.g., BMW, Audi AG, Porsche AG, and Volkswagen Group). Thus, OpenDRIVE is now being managed by an international board. OpenDRIVE provides a road evaluation library which can make the data exchange between different servers and applications easier. It is also available for vehicle dynamics, traffic simulation, and sensor simulation via the library.

As a complementary project, the OpenCRG project was established in October 2008 [30]. CRG stands for curved regular grid. Its objective is to provide open file formats and tools for the representation of high-precision 3D road surfaces. The predecessor of OpenCRG is a format called CRG, which has been used internally for several years by Daimler AG. OpenCRG is designed to represent road surfaces in very high resolution, so that the CRG files can be used for tire, vibration, and driving simulation.

**Figure 1:**
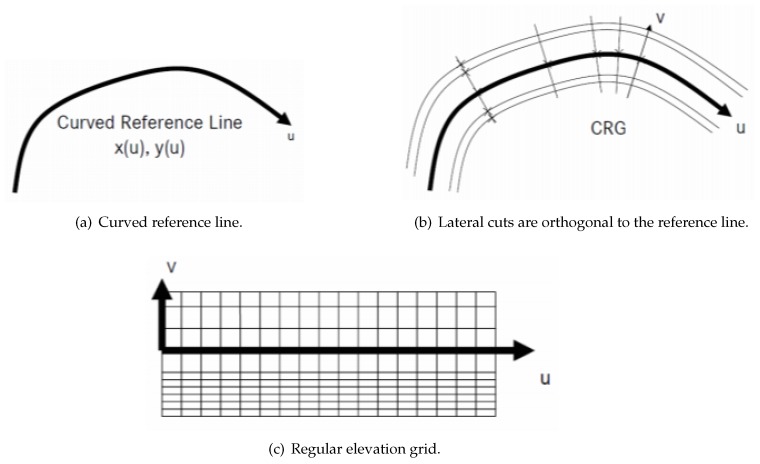
The basic idea of the curved regular grid (CRG).

In order to present the road data in a CRG model, the road parameters must be defined (e.g., start position, end position, road width, slope, heading angle, and elevation). Start position and end position are actually the start and end points of a road segment. As shown in Figure 1, a curved regular grid represents road elevation data close to a road center line. A CRG model consists of two major parts: a reference line and a regular elevation grid. The reference line is defined by a start position, an end position, and consecutive heading angles. The *u*-axis lies on the reference line and the *v*-axis is orthogonal to the reference line. The regular elevation grid is a special form of regular grid which is locally orthogonal. Columns are longitudinal cuts that are parallel to the reference line, and rows are lateral cuts that are orthogonal to the reference line. The grid nodes contain elevation information of the road surface. A MATLAB and C-API toolbox was developed for the handling, evaluation, and generation of CRG data.

In addition, the OpenSolutions family has expanded with a new member OpenSCENARIO [31]. It was formally presented in 2016 and is still in the development stage. The purpose of the project is to establish a standard for dynamic content in vehicle simulations, such as traffic models, driver models, infrastructure event models, etc.

### 1.2. Structure of the Paper

This paper is structured as follows. In Section 2, we give a brief review of our previous work. The proposed method is then described in detail. Experimental results are illustrated in Section 3, and Section 4 concludes this paper.

## 2. Method

### 2.1. Input Data Preparation

**Figure 2:**
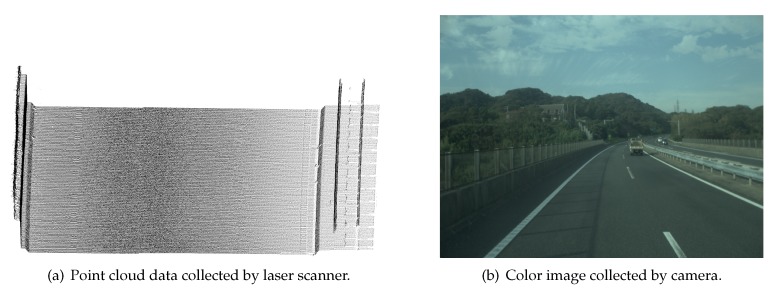
Dataset obtained by mobile mapping system.

The point cloud data and images used in our work were captured using a 3D laser line scanner and charge-coupled device (CCD) camera. The benefit of a 3D laser line scanner is that the vehicle position is known and can be used for information on the road’s location and orientation. Each point not only has three-dimensional coordinates, but also laser irradiation angle and GPS time. This information can be used to structurize the points. From this information, we use the laser irradiation angle in order to separate the point cloud into scan lines. Moreover, we order points in a scan line and find neighborhood elements by laser irradiation angle information. The point cloud data and color images used in our research are illustrated in Figure 2.

**Figure 3:**
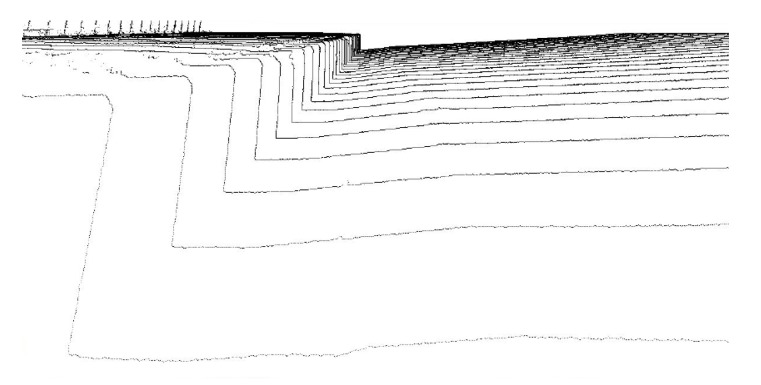
The difference of point density according to the direction.

However, the interval of measured points along the direction in which the MMS travels depends on the rotation speed of the laserirradiation part and the speed with which the MMS travels. The rotation period of the laser irradiation part is much longer than the laser irradiation period. The measurement interval along the direction of MMS travel is often about a few hundred millimeters. Thus, the density of the point distribution is greatly unbalanced with the direction (Figure 3).

This situation causes the problem in the method with creating regular grids of elevation values onto raw point cloud data directly. In such a case, a manual process is always involved, and this kind of method is very time-consuming. For example, in [32], the authors presented an algorithm for local gridding where the elevation values are computed based on local binning geometry. The accuracy and time complexity of this algorithm were not suitable for creating the CRG model.

In [27], in oeder to find the location of the boundary points precisely, we used a line-based region-growing method to extract the road surface region from the point cloud. Following this method, the input to our algorithm is a set of line segments. We first create line segments from a point sequence using the angle of laser irradiation. We then use the line segments as processing elements for the road surface region extraction. For searching neighborhood line segments, we use the laser irradiation angle associated directly with sampled points. If two points on two consecutive scanning lines have a similar laser irradiation angle, these points are considered to be located near each other. Next, we extract lane marks and their midpoints [25]. The road surface points are projected onto a color image to find the precise lane mark region. Then, we perform an inverse projection to recover the 3D coordinates of the detected 2D lane mark points. For the missing points between broken white lines, we describe the three-dimensional points in a length-angle space to fill the gap, considering that the road trajectories are sequences consisting of centerline points. Finally, we generate a 3D point sequence to represent the trajectory points [26], and hence we can use the trajectory of the road as the reference line in road surface modeling.

**Figure 4:**
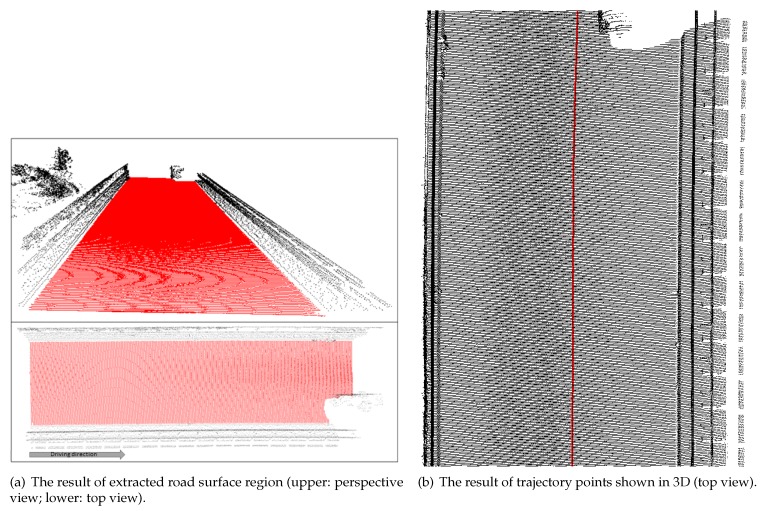
The results of our previous work.

Figure 4a,b shows examples of the results in our previous work. Figure 4a is an example of an extracted road surface region by line-based region growing. We painted the road surface points and non-road points in red and black, respectively. We can see that the line-based region growing method precisely extracted the road surface region. Figure 4b shows an example of 3D road trajectory points, which are colored in red.

### 2.2. Overview of the Method

In the first step, we divide the input trajectory point into three types: straight line, circular arc, and clothoid. Naturally, the curvature of a straight line is nearly zero, a circular arc has constant curvature, and the curvature of a clothoid varies linearly along the arc. The straight and circular road segments are both extracted by the RANSAC algorithm, which is not only able to give accurate detection result, but also maintain speed and stability. The straight lines are extracted first, then circular road segments are extracted from the rest of the data. Points between straight line and circular segments should be fitted by a clothoid curve.

**Figure 5:**
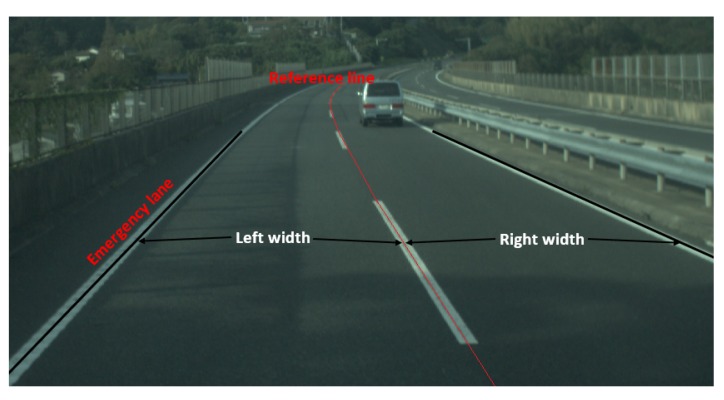
A road image of left width, right width, and emergency lane.

In the CRG model, the left and right road widths must be fixed. In the real world, there is an emergency lane on the side of the road, which means the width is not ideally equal. In our case, we are analyzing a symmetric road, which means that the width values are the same. Figure 5 illustrates the definitions of left width, right width, and emergency lane.

**Figure 6:**
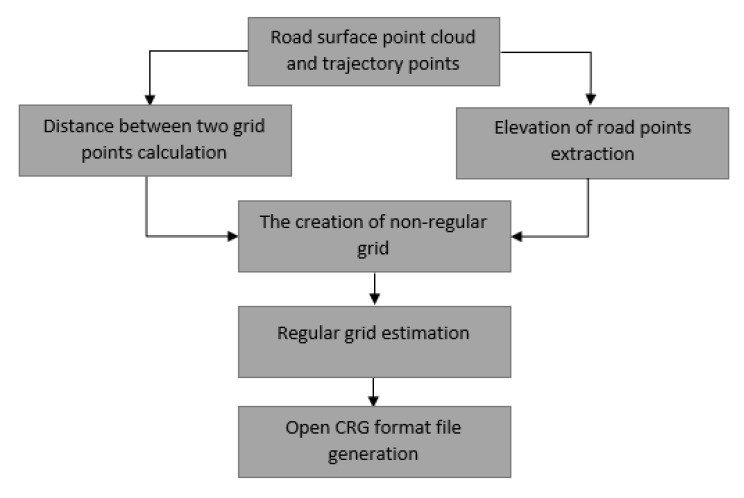
Processing workflow.

Elevation values are indicated by the z-values of each point. The regular grid of elevation is the most important component of the CRG file. Thus, we create a non-regular grid of elevation for each road segment in order to acquire a regular grid of elevation due to varied distance between points and sparse scanlines. A bilinear interpolation method is used here to compute the elevation of the regular grid point from surrounding non-regular grid points. Finally, we make the CRG file according to the format specification of OpenCRG model. The complete workflow used in this paper is illustrated in Figure 6.

### 2.3. Road Segmentation Process

To find straight lines and circular arcs, among many mature algorithms, the RANSAC algorithm is widely used because of its reliability and accuracy. Since the trajectory is a point sequence with continuous-curvature profile, we apply a common RANSAC strategy for straight line and circular arc detection. The *x* and *y* coordinates of trajectory points are used as input. A minimal subset of the trajectory points is chosen randomly and model parameters are estimated from this subset. The estimated model is then checked by the entire dataset and all data points are classified as inliers or outliers by calculating the residuals to the model. In each iteration, the algorithm performs the same actions until the best model is determined. The straight lines are first estimated and the circular arcs are detected from the remaining data points.

To deal with the transitions between road segments, we use the G2 interpolation method in [28] to estimate the clothoid curve. Given a start point $\left(x_{0},y_{0}\right)$ and an end point $\left(x_{1},y_{1}\right)$ of a transition clothoid curve, the heading angle and curvature are also calculated. In general, it is not possible to estimate a transition curve with only one clothoid. Considering two clothoid segments that have to join with G2 continuity, an intermediate point $M=(x_{M},y_{M})$ that joins the two arcs with a G2 hypothesis is introduced here. Assume the two segments are starting respectively from the start and the end point, matching at *M*. Given two points $\left(x_{0},y_{0}\right)$ and $\left(x_{1},y_{1}\right)$, two angles $\nu_{0}$ and $\nu_{1}$, and two curvatures $\kappa_{0}$ and $\kappa_{1}$, let $s_{0}$ and $s_{1}$ be the lengths of the two matching arcs, and the curvature change rates are $\kappa_{0}^{\prime }$ and $\kappa_{1}^{\prime }$. We can define two arcs by

(1) $$\begin{array}{c} s_{0}=\alpha L,s_{1}=\left(1-\alpha \right)L,\kappa_{0}^{\prime }=\frac{A(\alpha ,L)}{\alpha^{2}L^{2}},\kappa_{1}^{\prime }=\frac{A(\alpha -1,L)}{{\left(1-\alpha \right)}^{2}L^{2}}, \end{array}$$

where

(2) $$\begin{array}{c} A\left(\alpha ,L\right)=\alpha^{2}L\Delta \kappa +\alpha \left(2\Delta \nu -L\left(\kappa_{0}+\kappa_{1}\right)\right),\\ \Delta \kappa =\kappa_{1}-\kappa_{0},\Delta \nu =\nu_{1}-\nu_{0}, \end{array}$$

and α, *L* are the solution of the smaller nonlinear system:

(3) $$\begin{array}{c} \Delta x/L=\alpha X_{0}\left(A\left(\alpha ,L\right),\alpha L\kappa_{0},\nu_{0}\right)+\\ \left(1-\alpha \right)X_{0}\left(A\left(\alpha -1,L\right),\left(\alpha -1\right)L\kappa_{1},\nu_{1}\right) \end{array}$$

(4) $$\begin{array}{c} \Delta y/L=\alpha Y_{0}\left(A\left(\alpha ,L\right),\alpha L\kappa_{0},\nu_{0}\right)+\\ \left(1-\alpha \right)Y_{0}\left(A\left(\alpha -1,L\right),\left(\alpha -1\right)L\kappa_{1},\nu_{1}\right), \end{array}$$

where $\Delta x=x_{1}-x_{0}$ and $\Delta y=y_{1}-y_{0}$.

**Figure 7:**
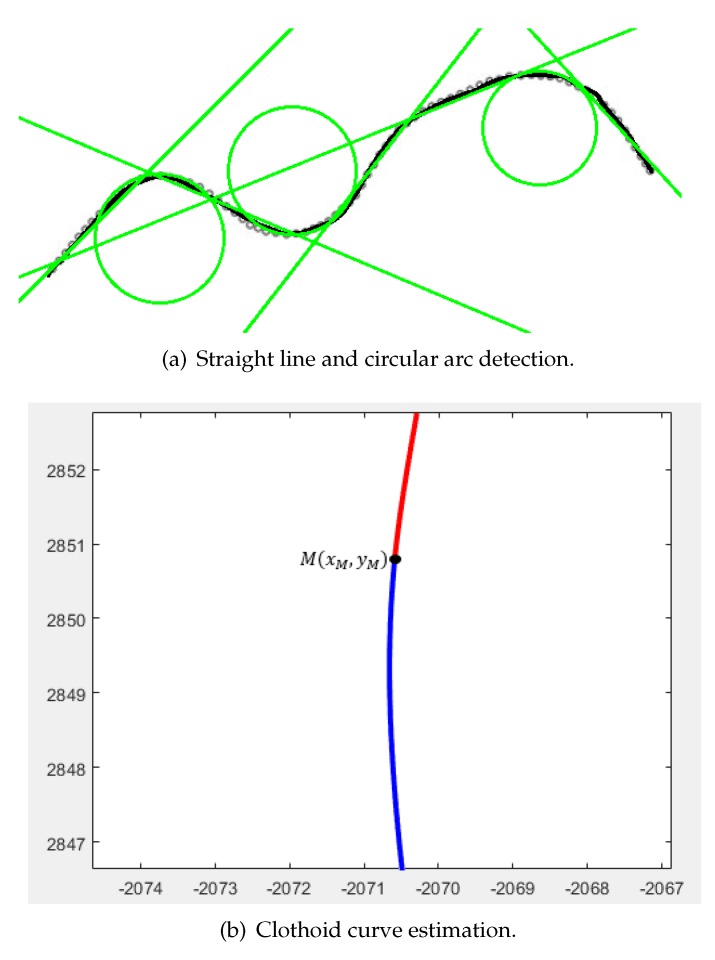
Example of straight line and circular arc detection (**top**), and clothoid curve estimation (**bottom**).

Figure 7 shows examples of finding straight lines, circular arcs, and clothoid curves. Figure 7a is an example of a RANSAC algorithm result. The example of clothoid estimation is shown in Figure 7b.

### 2.4. Creating a Non-Regular Grid

**Figure 8:**
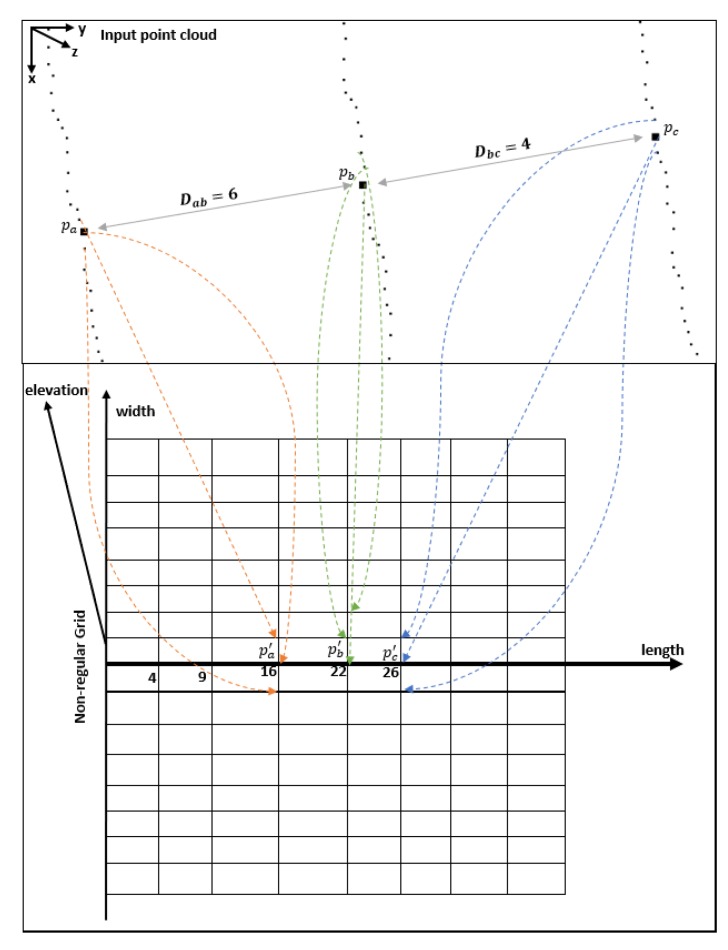
Approach for finding corresponding non-regular grid points (distance values in the figure are the unit distances).

After dividing trajectory points into road segments by three categories, to create a non-regular grid for each road segment, we separate the input point cloud into scanlines using laser irradiation angle, and we also find the ordering for the points on each scanline. Then, we assign a corresponding grid point in the non-regular grid for each point in the point cloud by the order of points on each scanline. Figure 8 shows the idea, where $p_{a}$, $p_{b}$, and $p_{c}$ are trajectory points, and horizontal and vertical distance values between measured points are adopted to non-regular grid points. For example, along both length and width axes, the distances between two grid points are calculated by two adjacent trajectory points and two neighbor points lying on the same scanline, respectively. In other words, horizontal distance means the length along the vehicle heading direction, and vertical distance means the width along the scanline. Based on the input road surface point cloud, we extract elevation by the *z*-value of each point.

### 2.5. Creating the Regular Grid

**Figure 9:**
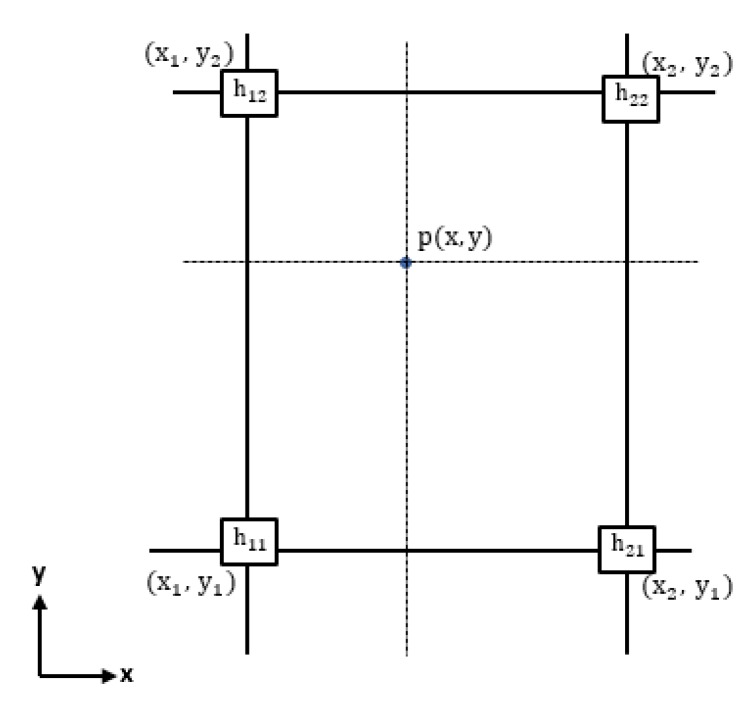
Bilinear interpolation.

According to the format specification of the OpenCRG model [33], in order to create an OpenCRG file, a regular grid which contains the elevation values of the points is needed. A bilinear interpolation is applied here to estimate a regular grid from a non-regular grid with a predefined resolution. In Figure 9 we illustrate the bilinear interpolation process.

For each interpolated point p(x,y), we find four surrounding non-regular grid points. The elevation value *h* at an interpolated regular grid point is defined as

(5) $$\begin{array}{c} h=\frac{1}{\left(x_{2}-x_{1}\right)\left(y_{2}-y_{1}\right)}(\left(x_{2}-x\right)\left(y_{2}-y\right)h_{11}+\\ \left(x-x_{1}\right)\left(y_{2}-y\right)h_{21}+\left(x_{2}-x\right)\left(y-y_{1}\right)h_{12}+\\ \left(x-x_{1}\right)\left(y-y_{1}\right)h_{22}), \end{array}$$

where *h* is the elevation at an interpolated point p(x,y), and $h_{11},\;h_{12},\;h_{21}$, and $h_{22}$ are the elevation values at four surrounding points.

### 2.6. Building CRG Model

Since the regular grid of elevation is created, we follow the specification of OpenCRG to make the file for each road segment. Start position, end position, and consecutive heading angles allow us to build a CRG model for the road data. Increments on reference line and spacing on the left and right of the reference line are both fixed by the user-defined resolution.

## 3. Results

In this paper, the input point cloud was measured by the MMS equipped with the Z+F IMAGER 5010 laser scanning system. The color images were taken by the same MMS during the data collection. The datasets were collected on a toll road in Yokohama, Japan. Considering the density of the input point cloud and [34], in order to find the straight line and circular arcs, the minimum and maximum length of a road segment were set to 20 m and 1000 m, respectively. The number of iterations of RANSAC was set to 2000 in order to ensure a reliable result.

### 3.1. Comparison of Non-Regular and Regular Grids

**Figure 10:**
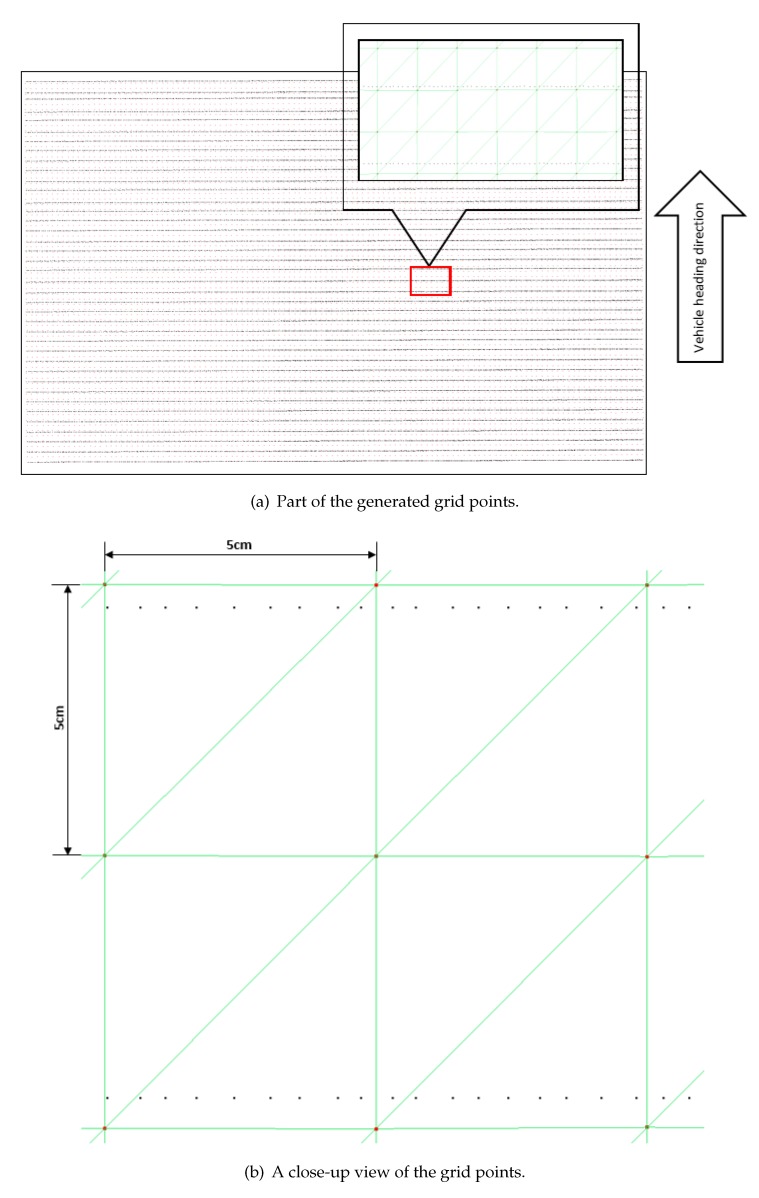
Comparison of non-regular and regular grids. Black dots are the non-regular grid points; red points are the estimated regular grid points.

The proposed method creates the non-regular grid first in order to find the regular grid. Considering that the expressway road is in good condition, a regular grid with a longitudinal and lateral resolution of 5 cm × 5 cm was estimated by our method. A magnified view of a sample of the generated non-regular and regular grids are shown in Figure 10. The black points represent the non-regular grid points in the road segment. The red points represent the estimated regular grid points. As can be seen, the spacing between non-regular grid points is uneven, and regular grid points were accordingly estimated from surrounding points. The elevation values of each grid were estimated from four optimal surrounding measured points by bilinear interpolation. To better observe the result, we created a mesh for the regular grid points. Figure 10b is a close-up view of the grid points, showing the relationship of two kinds of grid points. Consequently, the created regular grid can generate a uniform mesh, which means each grid cell has the same structure.

### 3.2. Building the CRG Model

**Figure 11:**
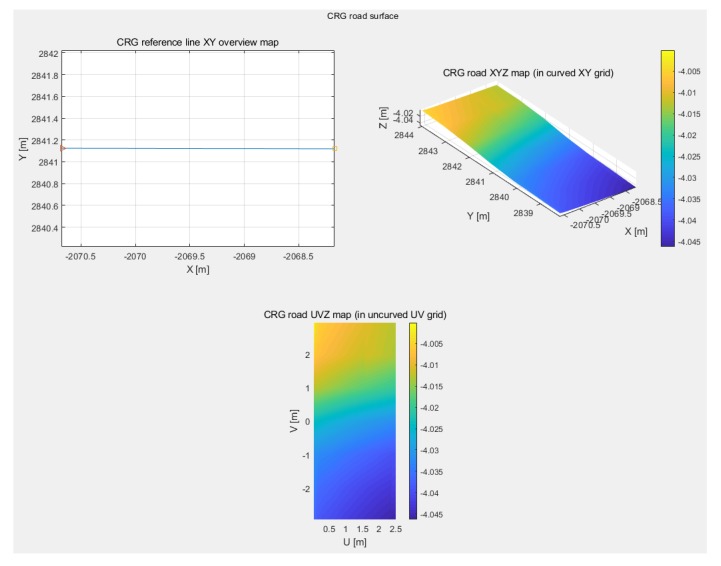
Example of a clothoid road segment visualization.

**Figure 12:**
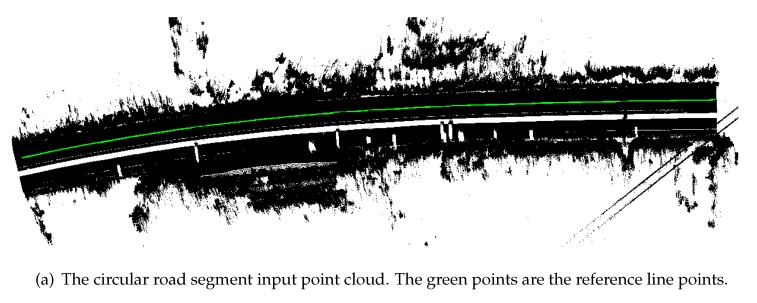

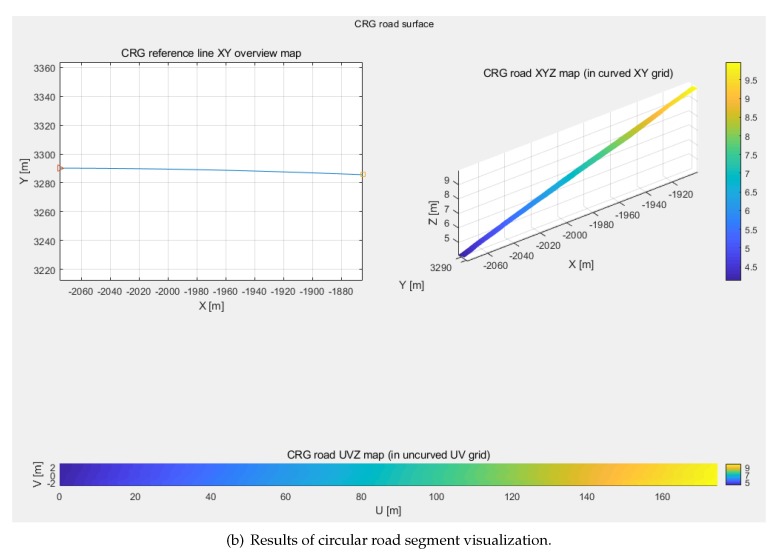
Example of a circular road segment visualization.

The CRG model was visualized by the OpenCRG MATLAB tools. The results consist of a reference line XY overview map, a road XYZ map, and a road UVZ map. The CRG road XYZ map represents the road segment in a curved XY grid with the Z axis as elevation. The road UVZ map shows the road in an uncurved UV grid with the Z axis as elevation. A 5 cm resolution CRG model was built in this experiment. Figure 11 provides an example of a clothoid road segment. The length of this clothoid segment was 2.5 m with 2.94 m width on both sides of the road center line, symmetrically. As we can observe clearly, the road had a slight height difference. The minimum height of the road was −4.04 m and the maximum height was −3.99 m. Here, the term “height” refers to the distance above (or below) mean sea level. The curvature changes were too small to see because the input data was obtained from a highway environment. Figure 12 illustrates a circular road segment CRG model. The length and width of the sample circular point cloud data were 173.45 m and 5.88 m, respectively. The height value changes are shown intuitively. The minimum and maximum height were 4.12 m and 11.08 m, respectively.

In our case, the generated regular elevation grid was denser than the input point cloud. It is difficult to evaluate the accuracy of the resultant model by numerical analysis, because there is no ground truth data. Since this method finds optimal measured points for estimation of the elevation of each grid by bilinear interpolation, the elevation values were estimated with reasonable accuracy. We assumed that the estimated elevation values by the proposed method were the ground truth data. One way to evaluate the effectiveness and accuracy is calculate the root-mean-square error (RMSE) to measure the differences of elevation values between the proposed method and a nearest-neighbor search (NNS) method. A subsampled grid was created from the non-regular grid by the distance between grid points. In our experiment, two subsets of points were obtained by assigning the distances of 0.05 m and 0.10 m. For each regular grid point $P_{i}$, we used the obtained elevation $Z_{i}$ as the reference elevation. The elevation $Z_{bi}$ of the point $P_{i}$ was calculated by the proposed method, and the closest grid point was used to obtain the elevation value $Z_{nn}$ by the NNS method. Then, we compared the elevation $Z_{bi}$ and $Z_{nn}$ with the reference elevation $Z_{i}$ and calculated the RMSE. The RMSE of residuals is defined by the following formula:

(6) $$\begin{array}{c} \mathrm{RMSE}=\sqrt{\frac{\sum_{i=1}^{n}{\left(Z_{ti}-Z_{i}\right)}^{2}}{n}}, \end{array}$$

where *n* represents the grid point number of the generated regular grid and $Z_{ti}$ is the elevation determined by the proposed method and the NNS method. The quantitative evaluation result is shown in Table 1.

Table 1 shows that the RMSEs of the 0.05 m spacing dataset for our method and the NNS method were 0.001062611 m and 0.005046622 m, respectively. The RMSE values for the 0.10 m spacing dataset were 0.001433533 m and 0.01131894 m, respectively. Our method achieved better accuracy on both datasets. In addition, it can be seen that the accuracy of the proposed method could maintain a high precision even when a sparse dataset was used as input.

The execution times for the two example CRG models were 4.75 s and 38.91 s on a PC with Intel Core i5-9600k at 3.70 GHz and 16 GB RAM. The preprocessing time cost for extracting the reference line by our previously presented method was less than 20 min. For comparison, a 259.55 m pilot site is used in the experiment in [23]. Their computational time for building the CRG model was 3 h. Since there is no detailed explanation on the composition of computational time, we assumed that the running time during the data preprocessing phase was also counted in this (e.g., manually finding the road surface region, manually finding the reference line, etc.).

## 4. Conclusions

Self-driving car techniques have advanced very quickly in recent years, and it is foreseeable that autonomous vehicles will become a common feature in the near future. Since autonomous vehicles have high demand on high-precision road models with detailed information about the surrounding environment, we propose an improved approach to create CRG models from mobile mapping data.

Based on our previous work, precise road surface region point cloud and trajectory are used to define the road surface. Therefore we can set accurate reference line parameters in the process of building the CRG model. To represent a hig-accuracy road surface model, a two-step approach was used to create the regular grid of elevation for the CRG model instead of creating a regular grid from the point cloud directly. The elevation values were more accurately estimated by using bilinear interpolation. The experimental results show that the proposed method could create a CRG model of the road in a very high resolution, and the resolution could also be customized. The visualized CRG model contains a microscopic view of the road surface. It could play an important role in the design and development of vehicles. Moreover, the proposed method is able to build road model without any manual intervention throughout the process. As a next step in future development, we may further build the road network database with the support of OpenCRG and OpenDRIVE, and hence we may establish a connection to the vehicle dynamics simulators.

## Figures and Tables

**Table 1:** Root-mean-square error (RMSE) of residuals in the proposed method and nearest-neighbor search (NNS) method.

Subsampling Spacing (m)	RMSE of the Proposed Method (m)	RMSE of NNS Method (m)
0.05	0.001062611	0.005046622
0.10	0.001433533	0.01131894
